# Intracellular matrix Gla protein promotes tumor progression by activating JAK2/STAT5 signaling in gastric cancer

**DOI:** 10.1002/1878-0261.12652

**Published:** 2020-03-16

**Authors:** Mizhu Wang, Lei Chen, Yu Chen, Rui Wei, Qingdong Guo, Shengquan Zhu, Shuilong Guo, Shengtao Zhu, Shutian Zhang, Li Min

**Affiliations:** ^1^ Department of Gastroenterology, Beijing Friendship Hospital, Capital Medical University National Clinical Research Center for Digestive Disease Beijing Digestive Disease Center Beijing Key Laboratory for Precancerous Lesion of Digestive Disease Beijing China; ^2^ Inner Mongolia Institute of Digestive Diseases The Second Affiliated Hospital of Baotou Medical College Inner Mongolia University of Science and Technology Baotou China

**Keywords:** gastric cancer, JAK2/STAT5 pathway, matrix Gla protein, progression

## Abstract

Matrix Gla protein (MGP) has been widely reported as an extracellular matrix protein with abnormal expression in various types of cancer. However, the function of intracellular MGP in gastric cancer (GC) cells remains largely unknown. Here, we demonstrated aberrantly high expression of intracellular MGP in GC as compared to adjacent normal tissues by immunohistochemistry. Moreover, The Cancer Genome Atlas (TCGA) dataset analysis suggested a positive correlation between MGP overexpression and unfavorable prognosis. MGP silencing reduced cell proliferation, migration, invasion, and survival in GC cell lines. Gene set enrichment analysis of TCGA dataset indicated significant enrichment of the IL2–STAT5 signaling in MGP‐high GC patients. Immunofluorescence staining and immunoprecipitation showed that MGP binds to p‐STAT5 in the nuclei of GC cells. Furthermore, ChIP‐qPCR and luciferase reporter assays indicated that MGP acts as a transcriptional co‐activator through the enhancement of STAT5 binding to target gene promoters. Use of STAT5 inhibitor revealed that the oncogenic functions of intracellular MGP mainly depend on the JAK2/STAT5 signaling pathway. Taken together, our results indicate that intracellular MGP promotes proliferation and survival of GC cells by acting as a transcriptional co‐activator of STAT5. The detected aberrant, high MGP expression in GC tissues highlights MGP as a potential new prognostic biomarker in patients with GC.

## Graphical abstract

Matrix Gla protein is a novel transcriptional co‐activator of STAT5 by interacting with phospho‐STAT5 in the nuclei, which facilitates STAT5 binding to target gene promoters and subsequently promotes gastric cancer cell proliferation, migration, invasion and survival.

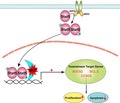

**Abbreviations**
BCL‐2: B‐cell lymphoma 2
BCL‐6: B‐cell lymphoma 6
CCND2: cyclin D2
DFS: disease‐free survival
GC: gastric cancer
GEO: Gene Expression Omnibus
GESA: gene set enrichment analysis
GLI1: glioma‐associated oncogene homolog 1
IGF1: insulin‐like growth factor 1
JAK2: Janus kinase 2
MGP: matrix Gla protein
OS: overall survival
p‐STAT5: phosphorylated signal transducer and activator of transcription 5
qRT–PCR: quantitative real‐time polymerase chain reaction
SOCS2: suppressor of cytokine signaling 2
STAT5: signal transducer and activator of transcription 5
TCGA: The Cancer Genome Atlas

## 1 Introduction

Gastric cancer (GC) is predicted to be the sixth most frequently diagnosed cancer and the second most common cause of cancer death worldwide, with over 1 000 000 new cases and 783 000 deaths occurring in 2018 (Bray *et al.*, 2018). The prognosis and therapeutic options of GC patients largely depend on clinicopathological parameters. Although many novel strategies for treating GC have been developed, the recurrence and metastasis rates are still incredibly high (Hartgrink *et al.*, 2009). Adequate surgery combined with adjuvant/neoadjuvant chemotherapy and radiotherapy remains the only possible curative way for nonmetastatic GC patients (Bang *et al.*, 2010; Hartgrink *et al.*, 2009; Macdonald *et al.*, 2001; Stahl *et al.*, 2015). Therefore, it is critical to explore the underlying molecular mechanisms of GC and find out new targets for drug development.

Matrix Gla protein (MGP) is a 12 kD secreted protein that contains five to six γ‐carboxyglutamic acid residues. MGP was originally isolated from bone tissue and has been considered to be involved in the inhibition of ectopic calcification of kidney, heart, cartilage, vascular smooth muscle cells (Mertsch *et al.*, 2009). Generally, MGP was considered as a secreted protein that could inhibit calcification by binding calcium ions (Price, 1989). Over the past a few decades, a growing number of studies have drawn attention to its role in tumor pathology.

Previous studies showed aberrant expression of MGP in various types of cancers and exert dual biological functions, acting as an oncogene or tumor‐suppressor, by regulating the expression of different target genes (Gheorghe and Crăciun, 2016). However, the regulatory role of MGP in tumorigenesis is still controversial. Overexpression of MGP was reported in glioma (Mertsch *et al.*, 2009), testicular carcinoma (Levedakou *et al.*, 1992), ovarian cancer (Hough *et al.*, 2001), and precancerous cervical lesions (de Wilde *et al.*, 2008). On the other hand, loss of MGP expression was identified in lung cancer (Bianchi *et al.*, 2004), suggesting a potential antitumor function. Surprisingly, there were opposite results reported in the same type of cancer. Yoshimura *et al.* (2009) suggested that MGP was upregulated in breast cancer and associated with poor prognosis. However, Daniel *et al*. provided evidence that miR‐155 promoted breast cancer cell proliferation and invasion by repressing MGP (Guo *et al.*, 2010), suggesting the tumor‐suppressive role of MGP. Similarly, overexpression of MGP was observed in primary renal and prostatic carcinomas (Levedakou *et al.*, 1992), while a loss of MGP expression in metastatic renal cell carcinoma and prostatic carcinoma as compared to the primary tumors was also reported (Levedakou *et al.*, 1992). In GC, the potential effects and relevant mechanisms of MGP remain largely uncovered.

JAK2/STAT5 pathway is crucial in many biological processes, including differentiation, proliferation, and apoptosis (Paukku and Silvennoinen, 2004). Abnormal expression and dysregulation of STAT5 were reported in different malignancies (Teglund *et al.*, 1998). It is also suggested that STAT5 could contribute to carcinogenesis by inhibiting cancer cell apoptosis (Nosaka *et al.*, 1999; Onishi *et al.*, 1998; Socolovsky *et al.*, 1999), promoting proliferation and cell cycle progression (Mori *et al.*, 1998; Nieborowska‐Skorska *et al.*, 1999). The JAK2/STAT5 pathway associated biological processes were mostly triggered by transcriptional activation of target genes of STAT5, such as BCL‐xL (Socolovsky *et al.*, 1999), PIM‐1 (Lilly *et al.*, 1992), SOCS family (Chen *et al.*, 2000; Davey *et al.*, 1999; Krebs and Hilton, 2000)*.* However, the crosstalk between MGP and the JAK2/STAT5 pathway has not been reported.

In this study, we investigated the expression of MGP in GC and normal tissues and revealed its correlation with clinical characteristics and prognosis. Bioinformatic analysis suggested a potential association between MGP and STAT5 signaling. Besides, the following biochemical assays demonstrated that MGP can suppress GC cell apoptosis by binding to the promoter of p‐STAT5 and subsequently activating the transcription of downstream genes.

## 2 Materials and methods

### 2.1 Patients and tissue specimens

A total of 71 GC tissues and pair‐matched adjacent normal tissues with complete clinicopathologic information were used for immunohistochemistry (IHC) staining. All specimens were obtained from the patients who underwent surgical resections and were diagnosed as GC in Beijing Friendship Hospital, Capital Medical University, China. All pathological diagnoses were confirmed by two different pathologists. This study was approved by the Ethics Committee of Beijing Friendship Hospital. Written informed consent was obtained from each participant. Detailed clinicopathologic parameters of GC patients involved in this study are exhibited in Table 1. The study methodologies conformed to the standards set by the Declaration of Helsinki.

**Table 1:** Associations between clinicopathological factors and MGP expression in 71 GC patients.

Variables	MGP		χ^2^	*P*‐value
	Positive (*n* = 52)	Negative (*n* = 19)		
Gender				
Male	37	16	2.0384	0.1534
Female	15	3		
Age (years)				
≥ 65	24	6	1.2115	0.2710
< 65	28	13		
Tumor size(cm)				
≥ 5	32	9	1.1451	0.2846
< 5	20	10		
T stage				
T1, T2	7	5	2.6802	0.1016
T3, T4	45	14		
Lymph node metastasis				
Negative	13	10	4.8513	0.0276
Positive	39	9		
Distant metastasis				
Negative	47	16	0.0927	0.7608
Positive	5	3		
Pathological stage				
I–II, II	16	4	0.6493	0.4204
II–III, III	36	15		
Clinical stage				
I, II	27	9	0.1155	0.7340
III, IV	25	10		

### 2.2 Immunohistochemistry

Paired tissue slides were deparaffinized through a series of xylene after incubated at 65 °C for 1 h and then rehydrated in graded alcohols. After antigen retrieval by high pressure, endogenous peroxidase activity was blocked with 3% H_2_O_2_ for 20 min. Sections were blocked by goat serum for 1 h and then incubated with diluted MGP monoclonal antibody (1 : 50, Cat No: SC‐81546; Santa Cruz, Dallas, TX, USA) overnight at 4 °C. The next day, after incubated with biotinylated anti‐mouse secondary antibody for 2 h at room temperature, peroxidase activity was detected by diaminobenzidine (DAB). Finally, following counterstaining with hematoxylin, IHC slides of paired normal and tumor tissues were scored based on the staining intensity of the cytoplasm and nucleus, respectively. The scores were estimated by two pathologists and divided into two parts: the staining intensity (0, negative; 1, weak positive; 2, moderately positive; and 3, strongly positive.) and the staining range (0, negative; 1, 1–33%; 2, 34–66%; and 3, 67–100%). The final scores were dependent on both of these two parts.

### 2.3 Cell culture and transfection

Two human GC cell lines, AGS and BGC‐823, were, respectively, purchased from American Type Culture Collection (ATCC, Manassas, VA, USA) and Chinese National Infrastructure of Cell Line Resource and were used to perform most of the experiments. Both of them are adherent cell lines derived from human epithelial gastric adenocarcinoma tissues but they have different origins. The AGS cell line was derived from fragments of a biopsy specimen of an untreated 54‐year‐old Caucasian woman according to ATCC. The BGC‐823 cell line originated from a 62‐year‐old Chinese patient with undifferentiated gastric adenocarcinoma according to the Chinese National Infrastructure of Cell Line Resource. Both of them are qualified cell lines widely used for in vitro experiments and are believed to be representative in GC studies. AGS cells were cultured in Ham's F‐12K (Kaighn's) medium (F‐12K; Gibco, Waltham, MA, USA) while BGC‐823 cells in Dulbecco's modified Eagle's medium (DMEM; Gibco) both supplemented with 10% FBS (Gibco) in a 37 °C humidified incubator with 5% CO_2_. Cell lines used in the experiments were performed no more than 10 passages.

Transfection of MGP siRNAs was conducted by using lipofectamine 3000 (Thermo Fisher Scientific, Waltham, MA, USA) until 80% confluence of GC cells after seeding. The sequences of MGP siRNAs (si‐MGP#1 and si‐MGP#2) and the scrambled siRNA (si‐Ctrl; Biolino Nucleic Acid Technology, Beijing, China) utilized in the study are listed in Table S1. To make the ectopic expression of MGP in AGS cell line, the full‐length ORF of MGP was cloned into LV5 and then packaged in lentivirus with a 3xFlag‐tag and eukaryotic resistance to purine (Suzhou GenePharma Co., Ltd., Suzhou, China). Stable transfection was selected for at least 1 week with puromycin.

### 2.4 Cell viability assay

Cell proliferation was estimated by one‐step MTS (3‐(4,5‐dimethylthiazol‐2‐yl)‐5‐(3‐carboxymethoxyphenyl)‐2‐(4‐sulfophenyl)‐2H‐tetrazolium; Promega, Madison, WI, USA) assay. Cells were seeded in complete media (with 10% FBS) at a density of 3000 cells per well in 96‐well plates after transfection, and 6 h later, the medium was changed to 1% FBS. MTS reagents were added into the wells at the time points of 0, 24, 48, and 72 h. Then, the cell viability was measured with enzyme‐labeled meter (SpectraMax M3; Molecular Devices, San Jose, CA, USA). Three independent experiments were performed.

### 2.5 Colony formation assay

Cells were plated in F‐12K or DMEM supplemented with 10% FBS at a density of 1000 cells per well in six‐well plates after transfection. The cells were incubated at 37 °C with 5% CO_2_ for 10–14 days. Thereafter, visible colonies were fixed and stained by 0.1% crystal violet, and the number of colonies was counted and analyzed. Three independent experiments were performed.

### 2.6 Wound healing assay

Cells were plated in six‐well plates to confluence. Three wounds were made in each well using a sterile pipette tip. After washing with PBS carefully to remove cellular debris, cells were cultured in pure medium without FBS. The migration index was pictured at the same location of wells at the time point of 0, 12, 24, 36, and 48 h, respectively. Three independent experiments were performed.

### 2.7 Migration and invasion assay

Migration assay was performed using 8‐mm‐pore transwell chambers without Matrigel (Corning Costar, Corning, NY, USA). Cells were seeded in the upper transwell chambers of 24‐well plate with pure medium without serum. At the same time, culture medium with 10% FBS was only prepared for lower chambers without cells, acting as a chemoattractant to amplify cell motility. Then, cells were allowed to migrate through the porous membrane for 24 h at 37 °C. After washed with PBS, cells adhering to the upper surface of the membrane were cleared out gently while the cells sticking to the lower surface were fixed by methanol. The nuclei of cells were stained with DAPI and photographed with fluorescence. The invasion assay was performed similarly except that transwell chambers with Matrigel were used. Three independent experiments were performed.

### 2.8 Apoptosis detection assay

Cells were digested and resuspended in D'PBS after transfection. Annexin V‐PE/7‐AAD staining kit (BD Biosciences, San Jose, CA, USA) was used for cell staining according to the manufacturer's instructions. The apoptosis rates of different groups were detected by FACS after 15‐min staining. Three independent experiments were performed.

### 2.9 Quantitative real‐time polymerase chain reaction

Total RNA was extracted by Trizol (Invitrogen, Karlsruhe, Germany) from cells of different groups firstly. Quantitative real‐time polymerase chain reaction (qRT–PCR) was performed using SYBR‐green Mix (Invitrogen) and 7500 Real‐Time PCR Systems (Applied Biosystems, Waltham, MA, USA) with cycle parameters listed as bellow: 94 °C for 2 min followed by 40 cycles of 94 °C for 15 s, then 56 °C for 20 s, 72 °C for 30 s, and finally 72 °C for 2 min. By analysis of melting curves, $2^{-\Delta \Delta C_{T}}$ was used to calculate the relative gene expression of qPCR. Primers used in this study were purchased from Sangon Biotech (Shanghai, China), and the specific sequences are listed in Table S2. Three independent experiments were conducted.

### 2.10 Immunoprecipitation and western blot

Proteins were extracted from cells by using lysis buffer (Hepes 50 µm, NaCl 150 µm, EDTA 1 mm, 1% Triton, 10% glycerol, protease inhibitor cocktail), followed by sufficient sonication and centrifugation of 13 800 ***g*** for 10 min at 4 °C. After that, protein A/G agarose and antibody were added and incubated on a rotator at 4 °C overnight. The beads were washed by 500 µL lysis buffer three times and then denatured at 99 °C for 10 min. Proteins were isolated by electrophoresis using 10% SDS/PAGE and then transfected to a polyvinylidene fluoride (Millipore, Burlington, MA, USA) membrane. 5% (w/v) milk (nonfat milk powder in TBST) was utilized to block nonspecific binding sites for 2 h at room temperature. After that, membranes were incubated with different primary antibodies at 4 °C overnight. All of the antibodies used in the study are listed in Table S1. Upon washed with TBST for six times, blots were incubated with corresponding secondary antibodies for 1 h at room temperature. Finally, blots were detected with an enhanced Chemiluminescence imaging system (Bio‐Rad, Hercules, CA, USA). Three independent experiments were conducted.

### 2.11 Immunofluorescence staining

Cells were seeded on sterile coverslips in six‐well plates, washed with PBS for three times, and fixed by 4% paraformaldehyde for 15 min. To rupture the membrane, cells were permeabilized with 0.25% Triton X‐100 in PBS, followed by blocking in 5% BSA in PBST for 1 h. Cells were incubated with primary antibody mixture (anti‐MGP 1 : 50, antiphosphorylated‐STAT5 1 : 50) at 4 °C overnight. After that, fluorescent secondary antibody mixture (Alexa Fluor 488 goat anti‐mouse IgG, 1 : 200; Life Technologies, Waltham, MA, USA; Alexa Fluor 594 goat anti‐rabbit IgG, 1 : 200; Life Technologies) were incubated with the cells for 2 h at room temperature. Finally, the cells were washed with PBS for three times and with DDW for one time. Nuclei were stained with DAPI and photographed by confocal microscopy (IX83, FLUOVIEW FV1200; Olympus, Tokyo, Japan).

### 2.12 Luciferase reporter assay

The pGL3 plasmids carrying SOCS2, BCL‐2, or CCND2 promoter regions (from −2000 base pairs to the transcription start site) and the luciferase reporter gene were purchased from YouBio, Changsha, China. The three plasmids were, respectively, transfected into GC cells using lipofectamine 3000 (Thermo Fisher Scientific). After 48‐h transfection, the cells were lysed and luciferase activity values were measured with the Dual‐Luciferase Reporter Assay System (Promega).

### 2.13 ChIP assay

ChIP assay was performed using the Magna ChIP G Assay Kit (Millipore) according to the manufacturer's instructions. Cells were cross‐linked with 37% formaldehyde, pelleted, and resuspended in lysis buffer, then sonicated and centrifuged to remove the insoluble material. The supernatants were collected and incubated overnight with indicated antibodies and Protein G magnetic beads. The beads were washed, and the precipitated chromatin complexes were collected, purified, and de‐cross‐linked at 62 °C for 2 h, followed by incubation at 95 °C for 10 min. The precipitated DNA fragments were subsequently quantified by qRT–PCR analysis. Specific sequences of SCOC2, BCL‐2, and CCND2 are shown in Table S3.

### 2.14 Statistical analysis

All data were analyzed using graphpad prism 5 (GraphPad Software, San Diego, CA, USA). The quantitative data obtained from the experiments are presented as the mean (±SD) unless otherwise specified. The significance of differences between two groups was estimated by Student's *t*‐tests, and multiple groups were estimated with one‐way ANOVA. Chi‐square test was used to analyze the associations of MGP expression with clinicopathological features. Kaplan–Meier plots and log‐rank tests were used to examine the differences in overall survival (OS) and disease‐free survival (DFS) between subgroups. *P* < 0.05 was considered statistically significant. Each biochemical and cellular experiment was conducted in triplicate unless otherwise specified.

## 3 Results

### 3.1 Aberrant high expression of MGP predicted poor prognosis in GC patients

**Fig. 1:**
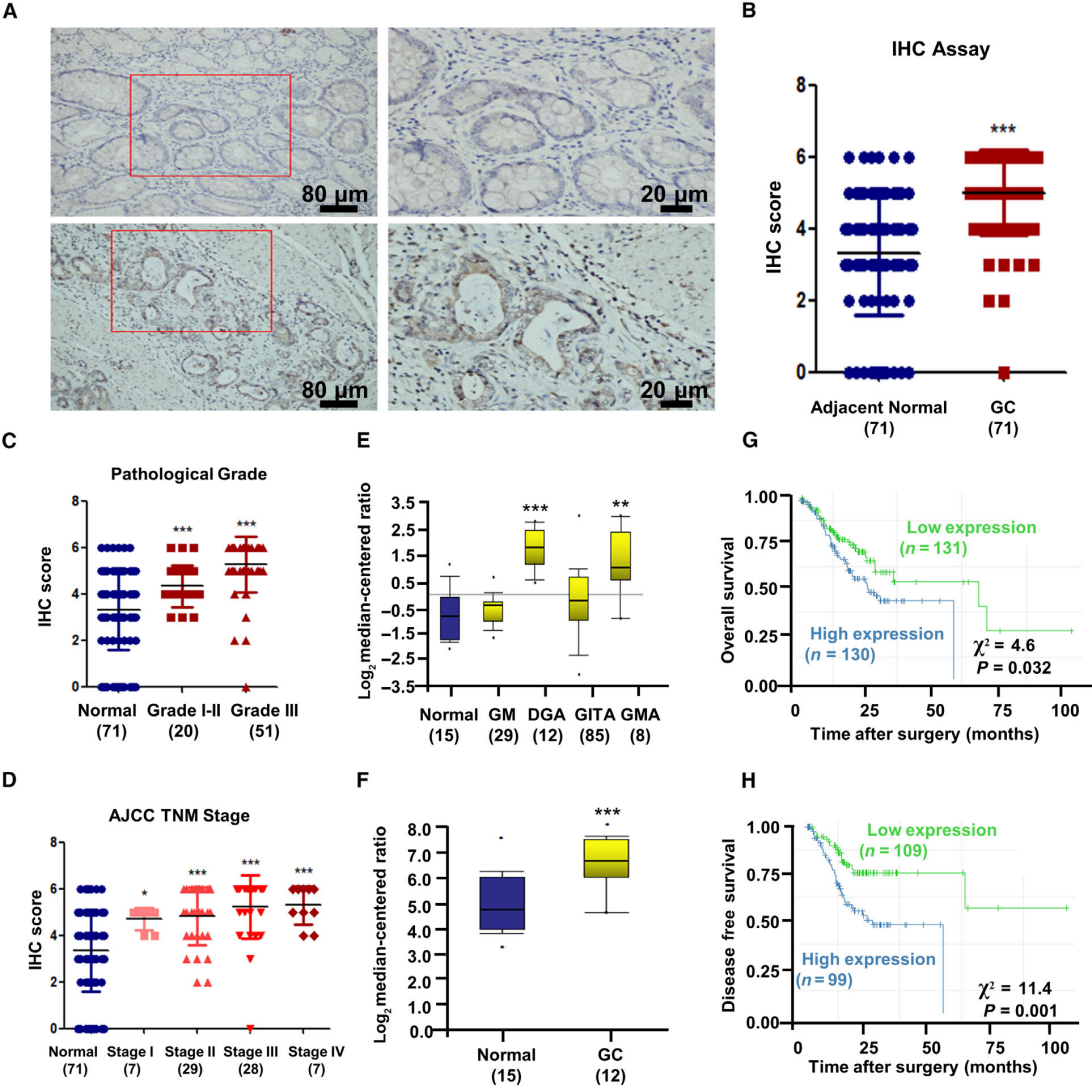
Matrix Gla protein expression was upregulated in GC and associated with poor prognosis. (A) GC and adjacent normal tissues were immunochemistry stained by 1 : 50 MGP antibody. GC tissues showed strong‐positive staining, while normal tissues indicated low MGP expression. (B) Analysis of IHC staining scores between 71 paired GC and normal tissues. The bars represent the lower quartile, median, and upper quartile. *P* value is estimated by Kruskal‐Wallis rank test. (C, D) IHC staining scores of GC at different pathological grades and four different AJCC TNM stages were analyzed and compared between normal and GC tissues. The bars represent the lower quartile, median, and upper quartile. *P *value is estimated by Kruskal‐Wallis rank test (E, F) MGP expression was different between normal gastric tissues and GC in mRNA level depending on a previously published GC dataset regarding variation in gene expression patterns (http://genome-www.stanford.edu/gastric_cancer2/index.shtml) and a GEO dataset (http://www.ncbi.nlm.nih.gov/geo/query/acc.cgi?acc=GSE19826). The results are presented as log2 median‐centered ratio. *P* values are estimated by one‐way ANOVA. (G, H) The OS (G) and DFS (H) of GC patients stratified by MGP expression levels. Kaplan–Meier plots and log‐rank tests were used for statistical analysis. *P < 0.05; **P < 0.01; ***P < 0.001.

Seventy‐one GC tissues and their paired adjacent normal tissues were used for IHC staining. We found that MGP protein mainly localized at nuclei and partially in cytoplasm of GC cells (Fig. 1A). The expression of MGP was significantly high in GC tissues as compared to that in the normal tissues (Fig. 1B, *P* < 0.001). We further evaluated the distribution of MGP expression in different pathological grades and in different TNM stages. MGP was significantly overexpressed in pathological grade I/II and grade III GC tissues as compared to the normal tissues (Fig. 1C, all *P* < 0.001). We also observed significant upregulation of MGP in TNM stage I to stage IV GC tissues in comparison with normal specimens (Fig. 1D, normal vs. TNM stage I, *P* < 0.05, all others *P* < 0.001). However, there is no statistical difference among different TNM stages (all *P *> 0.05), indicating MGP upregulation may be an early event in GC tumorigenesis. In addition, two independent public datasets supported high mRNA levels of MGP in GC as compared to non‐GC tissues (Chen *et al.*, 2003; Wang *et al.*, 2012) (Fig. 1E,F).

To further investigate the potential prognostic value of MGP, we performed survival analysis in TCGA (The Cancer Genome Atlas) database (Ooi *et al.*, 2009). MGP‐high GC cohort showed a worse prognosis in both OS (Fig. 1G) and DFS (Fig. 1H), compared to MGP‐low GC cohort. Analysis of other Gene Expression Omnibus (GEO) datasets further verified the prognostic value of MGP in GC patients (Fig. S1).

### 3.2 MGP promoted cell proliferation, migration, and invasion and inhibited apoptosis in GC cell lines

**Fig. 2:**
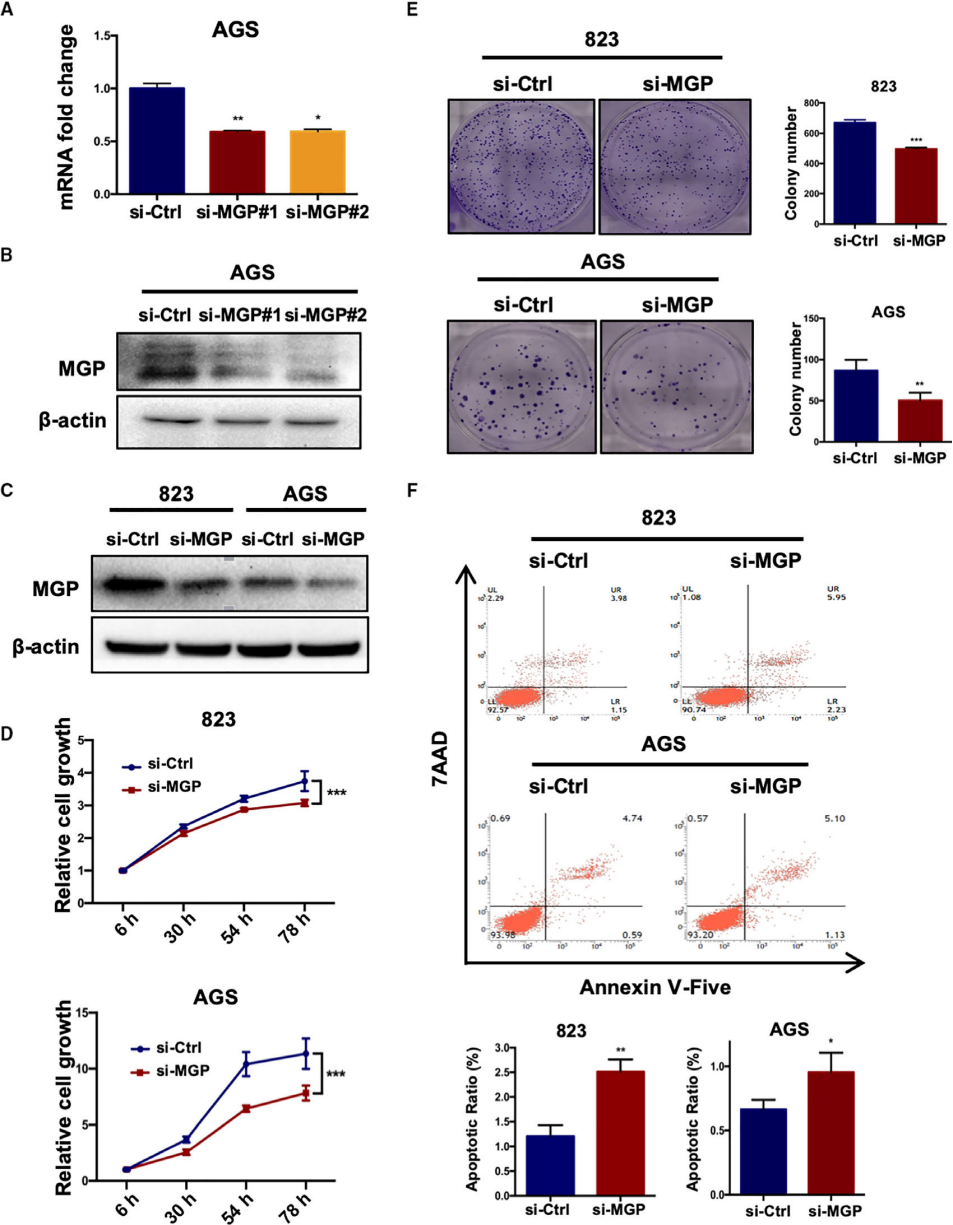
Knockdown of MGP inhibited cell proliferation and promoted apoptosis of GC cell lines. (A, B) Both MGP mRNA and protein levels were knocked down by two independent siRNAs in BGC823 and AGS cell lines. qRT–PCR results are presented as fold changes based on log2 values normalized to GAPDH. *P* values are estimated by one‐way ANOVA. (C) The MGP‐siRNA1 exhibited better knockdown efficiency and was selected for the following experiments. (D) Growth curves of BGC823 and AGS cells treated with MGP siRNA (si‐MGP) or scramble siRNA (si‐Ctrl). *P* values are estimated by Student's *t*‐tests. (E) Colony formation assay of BGC823 and AGS cell lines. *P* values are estimated by Student's *t*‐tests. (F) Apoptosis rate detected by FACS after MGP knockdown in BGC823 and AGS cell (upper panel: representative FACS scatter plots, up, BGC823; down: AGS; lower panel: statistics of early apoptosis cell percentages). *P* values are estimated by Student's *t*‐tests. *P < 0.05; **P < 0.01; ***P < 0.001.

To investigate the biological functions of MGP in GC, we performed MGP knockdown in BGC823 and AGS cell lines. RT–qPCR and WB examined the knockdown efficiency of two different siRNAs for MGP (Fig. 2A,B). The si‐MGP#1 was used for the following experiments due to the better knockdown efficiency (Fig. 2C). Interestingly, effective MGP knockdown remarkably reduces cell viability and proliferation of GC cells (Fig. 2D,E) by MTS and colony formation assays. Meanwhile, MGP knockdown significantly induced more early apoptosis in BGC823 and AGS cells (Fig. 2F).

**Fig. 3:**
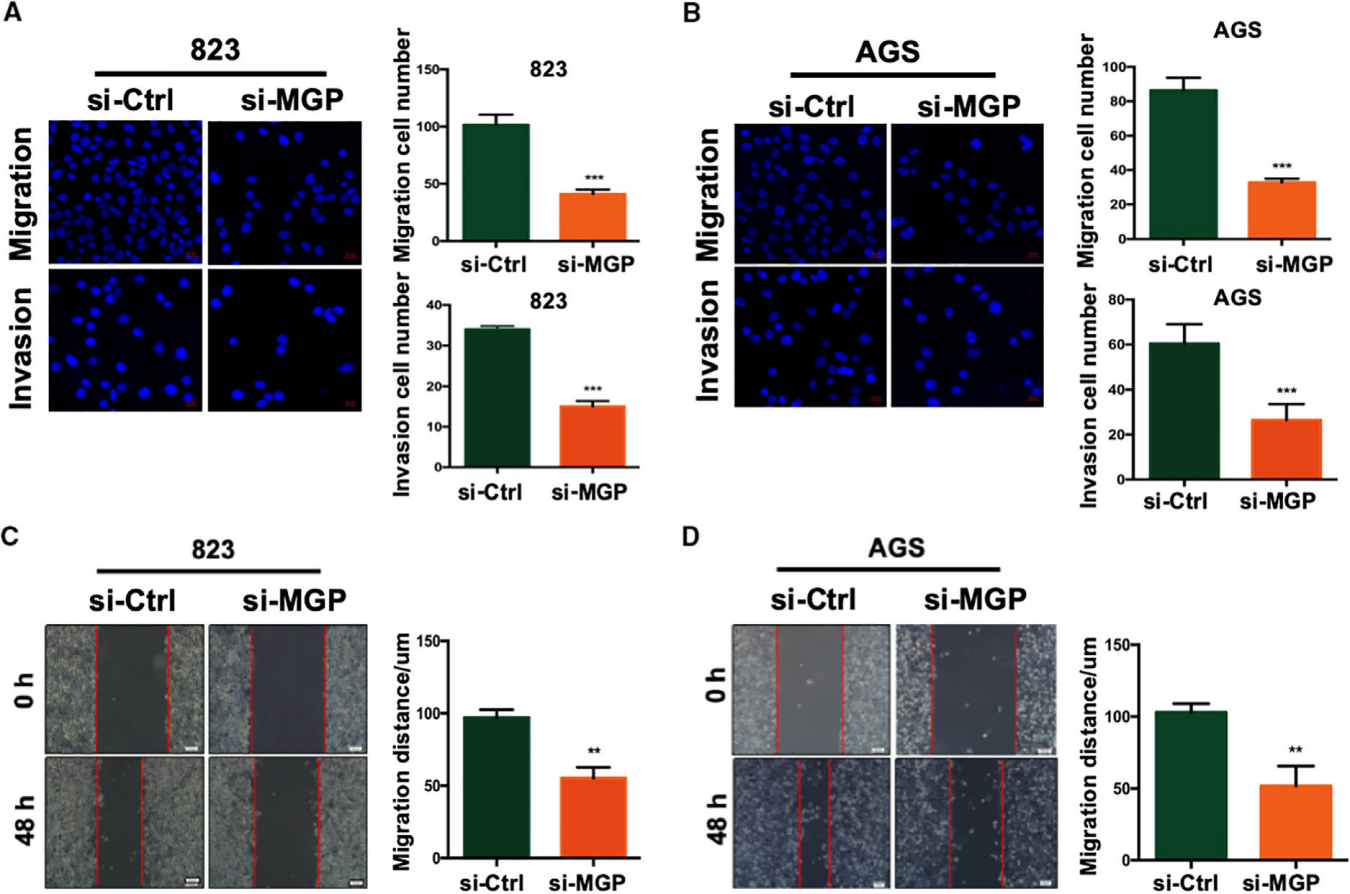
Matrix Gla protein promoted GC cell migration and invasion. (A, B) Migration (up panel) and invasion (down panel) transwell assays in BGC823 (A) and AGS cell lines (B) with MGP knockdown. Cells transferred to the other side of the chamber membrane were stained with DAPI and counted. Results are representative of three independent experiments. Values are the mean ± SD of the results. *P* values are estimated by Student's *t*‐tests. (C) Wound healing assays in BGC823 (C) and AGS (D) cell lines with MGP knockdown. Migrated distance was recorded at the time point of 36 h and analyzed in three independent experiments. *P* values are estimated by Student's *t*‐tests. **P < 0.01; ***P < 0.001.

Next, we examined effects of MGP on GC cell mobility by transwell assays. siRNA knockdown of MGP significantly decreased the migration and invasion abilities in both BGC823 and AGS cells (Fig. 3A,B), compared to the control siRNA. Wound healing assays also confirmed that MGP is important for GC cells mobility (Fig. 3C,D).

### 3.3 MGP interacted with p‐STAT5 in GC cells

**Fig. 4:**
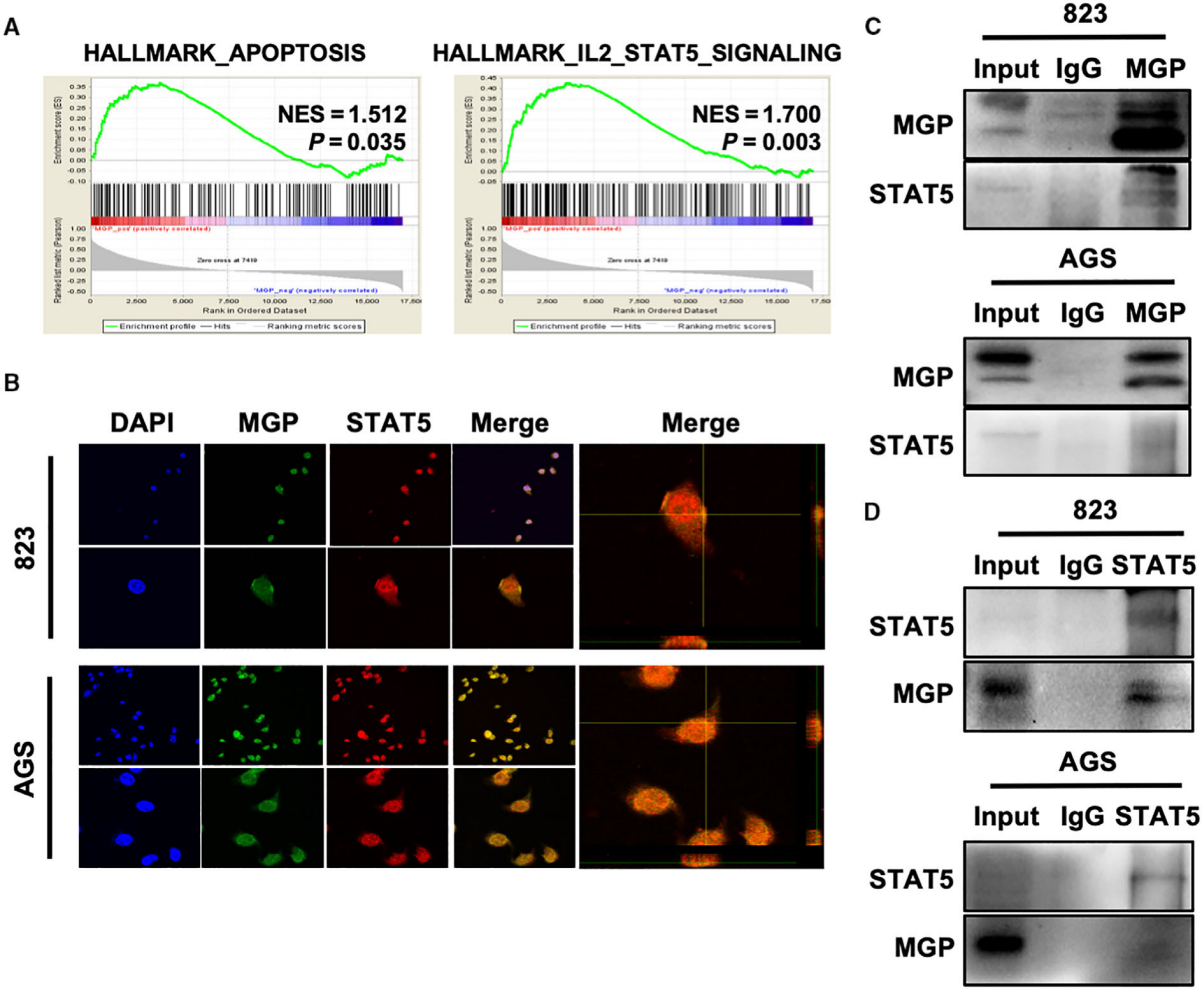
Matrix Gla protein interacted with p‐STAT5 in GC cells. (A) GESA analysis indicated that the expression level of MGP was positively correlated with both hallmark of apoptosis (left) and hallmark of IL2/Stat5 signaling (right). (B) Cellular immunofluorescence colocalization detected by confocal microscopy in BGC823 (upper panel) and AGS (lower panel). MGP (green) and STAT5 (red) pefectly colocalized with each other. DAPI was used to stain nuclei, and magnified 3D projection was displayed in the right panel. (C, D) Co‐IP assays were performed in BGC823 and AGS cells with both MGP (two above) and STAT5 (two below) antibodies, which indicated a strong interaction between MGP and STAT5. Three samples were pipetted into three different lanes according to the antibodies added or not added to the samples (input group: no antibody; IgG group: IgG; MGP group: anti‐MGP antibody; STAT5 group: anti‐STAT5 antibody). The protein levels of MGP and STAT5 were detected by western blot analysis.

To reveal the underlying mechanism contributing to the oncogenic effects of MGP, we performed gene set enrichment analysis (GESA) in all GC patients of TCGA datasets. The results demonstrated that apoptosis and IL2/STAT5 signals are significantly enriched in MGP‐high GC tissues, compared to MGP‐low tissues (Fig. 4A, *P* = 0.035, and *P* = 0.003, respectively). Therefore, we proposed that MGP promote GC progression by activating JAK2/STAT5 signaling. Since IHC assays discovered the nuclear localization of MGP in GC tissues, we suggested there could be an interaction between MGP and transcriptional factor STAT5 in nucleus. We confirmed nuclear colocalization of MGP and STAT5 by immunofluorescence costaining (Fig. 4B) in GC cell lines. Additionally, IP assays verified that endogenous MGP interacted with STAT5 in BGC823 and AGS cells (Fig. 4C,D).

### 3.4 MGP promoted expression of STAT5‐regulated genes

**Fig. 5:**
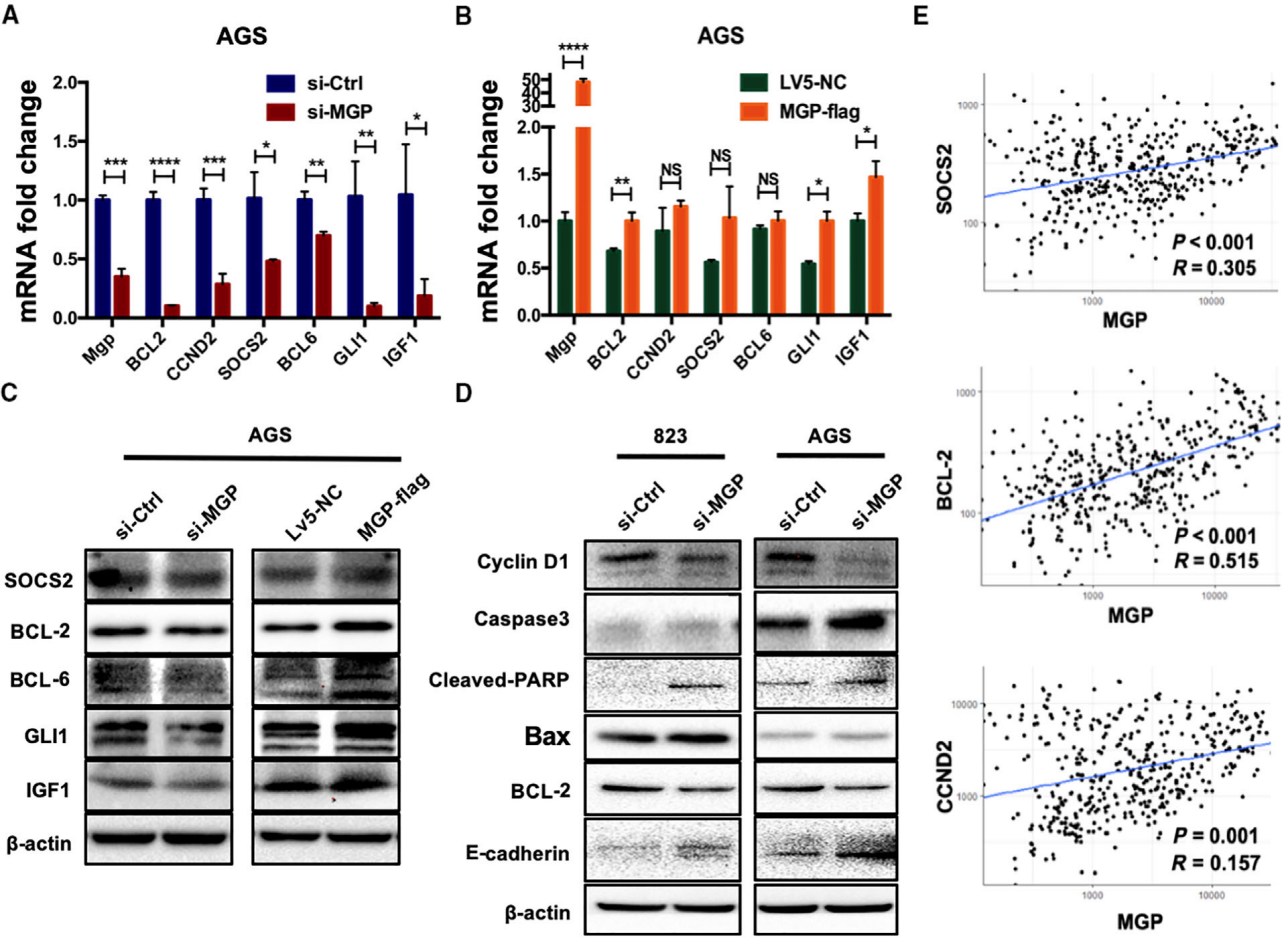
Matrix Gla protein elevated the expression of STAT5 downstream genes. (A, B) MGP siRNA knockdown decreased the mRNA expression level of STAT5 downstream genes (left), while MGP overexpression significantly upregulated these downstream genes (right) in AGS cells. The detected genes by qRT–PCR included BCL‐2, CCND2, SOCS2, BCL‐6, GLI1, and IGF1. The results are presented as fold changes based on log2 values normalized to GAPDH. *P* values are estimated by Student's *t*‐tests. (C) MGP siRNA knockdown decreased the protein level of STAT5 downstream genes (left panel), while MGP overexpression elevated the downstream genes (right panel) in AGS cells. (D) Knockdown of MGP upregulated the indicators of apoptosis and DNA damage, such as caspase 3, cleaved‐PARP, Bax, in both BGC823 (left panel) and AGS (right panel) cell lines. (E) Correlation analysis further confirmed the positive relationship between MGP and STAT5 target genes (upper: SOCSO2, middle: BCL‐2, lower: CCND2). *P < 0.05; **P < 0.01; ***P < 0.001; ****P < 0.0001.

To further investigate the effects of MGP on JAK2/STAT5 signaling, we examined the expression levels of several known downstream genes of STAT5 by RT–qPCR, including BCL‐2, CCND2, SOCS2, BCL‐6, GLI1, and IGF1 (Paukku and Silvennoinen, 2004). These genes were significantly downregulated by MGP knockdown and upregulated by MGP overexpression (Fig. 5A,B). Western blots also confirmed that BCL2, SCOS2, BCL6, GLI1, and IGF1 proteins were decreased by MGP knockdown and increased by MGP overexpression (Fig. 5C). Furthermore, we tested a series of apoptosis and DNA damage/repair associated proteins such as caspase 3, cleaved‐PARP, and Bax with MGP knockdown. We found that MGP knockdown decreased BCL2 and activated apoptotic signaling cascades (Fig. 5D).

We also analyzed the expression correlation between MGP and target genes of STAT5 in GC patients in TCGA datasets. Significant positive correlations were verified among MGP and STAT5 downstream genes including SOCOS2, BCL‐2, CCND2 (Fig. 5E), BCL‐6, IGF1, ESR1, and GLI1 (Fig. S2).

### 3.5 MGP acts as a transcriptional co‐activator of STAT5

**Fig. 6:**
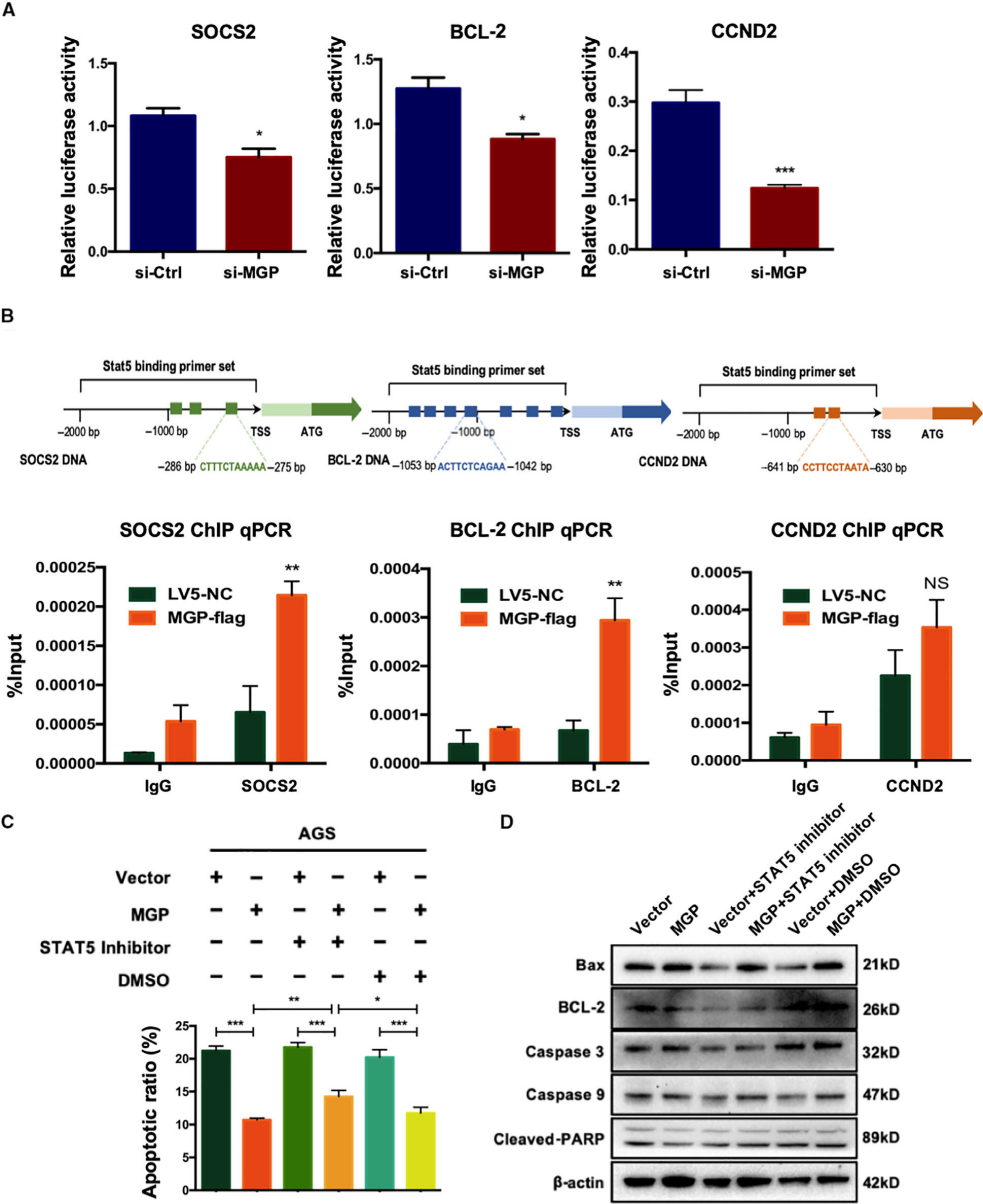
Matrix Gla protein promoted the transcription activation of STAT5. (A) Luciferase reporter assays of AGS cells that were cotransfected with MGP siRNA and pGL3 cloned with promoter regions of the three main STAT5 targets (left: SOCS2, middle: BCL‐2, right: CCND2). The results suggested that MGP knockdown induced suppression of the promoter activities of the three STAT5 target genes. *P* values are estimated by Student's *t*‐tests. (B) ChIP assays were performed in AGS cells transfected with si‐MGP or si‐Ctrl. MGP overexpression enhanced the transcriptional activation of the promoters of SOCS2 (left), BCL‐2 (middle), and CCND2 (right) through STAT5. Upper: schematic figures illustrated the locations of primer sets predicted online (http://jaspar.genereg.net/) in the promoter regions. Each pair of primers was tested in qPCR to detect the amplification efficiency. We selected the primers with the best quality in the following experiments. Lower: semiquantitative analysis of DNA fractions after ChIP (normalized by input, %). *P* values are estimated by Student's *t*‐tests. All assays were performed in triplicate, and one representative result is displayed. *P < 0.05; **P < 0.01. (C) Apoptosis rate detected by FACS in MGP overexpression AGS cells with or without STAT5 inhibitor treatment. The results showed that the apoptosis rate was partially reversed in MGP overexpressed AGS cells by STAT5 inhibitor. *P* values are estimated by one‐way ANOVA. (D) The protein levels of apoptosis/DNA damage‐associated genes, including Bax, caspase 3, caspase 9, cleaved‐PARP, and BCL‐2, were verified by western blot in MGP‐overexpressed AGS cells with or without STAT5 inhibitor treatment. The results supported the conclusion with reversed protein levels of apoptosis‐related genes after STAT5 inhibitor treatment. NS, no significance; *P < 0.05; **P < 0.01; ***P < 0.001; ****P < 0.0001.

To identify whether MGP affects STAT5 transcriptional activity, we performed luciferase reporter assays. MGP knockdown significantly suppressed the activation of the promoter of SOCS2, BCL2, and CCND2 (Fig. 6A).

ChIP‐qPCR further examined the effects of MGP on the direct binding of STAT5 to its target promoter regions. Our results showed that MGP overexpression significantly increased the DNA binding of STAT5 in the promoter regions of SOCS2, BCL2, and CCND2 (Fig. 6B). Taken together, we suggested that MGP acts as a transcriptional co‐activator of STAT5.

### 3.6 JAK2/STAT5 signaling is required for the anti‐apoptotic effects of MGP in GC

To investigate whether JAK2/STAT5 signaling is required for MGP‐mediated anti‐apoptotic functions in GC cell, a STAT5 inhibitor (Cat No: SC‐355979; Santa Cruz) was used to treat the cells combined with MGP overexpression. We found that STAT5 inhibitor partially abrogated MGP‐mediated anti‐apoptotic effects in AGS cells (Fig. 6C). Western blots also confirmed the effects of STAT5 inhibitor by regulating protein levels of apoptosis‐related genes such as BCL‐2, Bax, caspase 9, caspase 3, and cleaved‐PARP (Fig. 6D). Those results identified that MGP regulates anti‐apoptotic functions in GC in a JAK2/STAT5‐dependent manner.

## 4 Discussion

In this study, we found a significantly elevated MGP expression in GC at both mRNA and protein levels, which also indicated a poor prognosis. Interestingly, for the first time, we discovered the intracellular accumulation of MGP in GC cells, besides the known extracellular localization of MGP as previously reported (Hao *et al.*, 2004). Furthermore, we elucidated that MGP as a novel transcriptional co‐activator of STAT5, promoting GC cell proliferation, migration, invasion, and anti‐apoptosis. To our knowledge, our study is the first to report intracellular functions of MGP in cancer cells. Most previous studies of MGP were mainly focused on its extracellular biological functions, regardless of the fact that MGP also localized in the cytoplasm as previously reported (Guo *et al.*, 2010). Here, we confirmed the intracellular localization of MGP in GC cells and further revealed the intracellular regulatory role of MGP as a transcriptional co‐activator of STAT5. Our study also offered a possibility to explore the transcriptional regulatory functions of MGP for other transcription factors. Moreover, several previous studies demonstrated cross‐linking regulatory effects of calcium signal on STAT5 pathway (Chi *et al.*, 2016); however, the mechanism is not clear. MGP is known to have a strong calcium‐binding capacity, which suggests MGP may regulate STAT5 transcriptional activity by mediating calcium binding to MGP‐STAT5 complex at the promoter regions of downstream target genes. Our further study in the future will focus on examining this potential mechanism in cancer cells.

Previous studies found that activation of PI3K/AKT signaling increases MGP expression (Mirsaidi *et al.*, 2017; Ponnusamy *et al.*, 2018). Although the crosstalk of JAK2/STAT5 signaling and PI3K/AKT signaling has been reported (Britschgi *et al.*, 2012; Khanna *et al.*, 2018), the underlying mechanisms remain largely unknown. Our study used multiple lines of evidences supporting MGP as a novel transcriptional co‐activator of STAT5. This novel function of nuclear MGP suggests that MGP may act as a potential signaling transducer in the crosstalk of PI3K/AKT signaling and JAK2/STAT5 signaling.

Clinicopathological analysis of GC patients' samples and *in vitro* results suggested that MGP was not only a promising prognosis factor in GC patients, but also a critical regulator in the controlling of the JAK/STAT pathway. The unfavorable prognosis indicated by higher MGP levels could also be examined by a potential higher STAT5 signaling activity. However, we noticed that that MGP levels might not reflect TNM stage I–IV. Almost all independent prognostic factors are not associated with TNM stage, since the factors strongly associated with TNM stage will be eliminated from the Cox model in the backward process. Considering that TNM stage itself performs quite well in prognosis, only the independent prognostic factors which can be integrated with TNM stage in the same model are valuable. Biologically, the stable expression of MGP in GC of different stages suggested that the overexpression of MGP could occur very early during the carcinogenesis, but not in every case. GC is a highly heterogeneous disease, and different GC tumors have different genetic bases, which are determined at a very early stage. Thus, it is also reasonable that a protein expressed at an early stage could be a prognostic factor. In our study, the patients with higher MGP expression levels exhibited a worse prognosis than others, which is consistent with the tumor progression‐promoting role of MGP suggested in our in vitro studies by using GC cell lines.

The activation of JAK2/STAT5 pathway has been reported in different types of cancers. JAK2/STAT5 signal also was regarded as a promising drug target. Ruxolitinib, a potent inhibitor of JAK1/2, was approved by the FDA in the treatment of polycythemia vera and myelofibrosis. The application of ruxolitinib in the treatment of cancers is in phase II trials (Aittomäki and Pesu, 2014). Another potent JAK1/2 inhibitor, momelotinib, has been used to treat myeloproliferative neoplasms (Groner and von Manstein, 2017). Here, we also found STAT5 inhibitor could partially inhibit the oncogenic effects of MGP. Therefore, the GC patients with high MGP expression may be sensitive to STAT5 inhibitor treatment, which provided a potential precision medicine strategy by using STAT5 inhibitor combined with other chemotherapy in MGP‐high GC patients.

## 5 Conclusions

Matrix Gla protein promotes GC cell proliferation, migration, invasion, and survival. MGP interacts with STAT5, activating the transcription of the JAK2/STAT5 signaling components, which suggests MGP as a novel transcriptional co‐activator of STAT5. Aberrant intracellular expression of MGP in GC highlights MGP as a potential new prognostic biomarker.

## Conflict of interest

The authors declare no conflict of interest.

## Author contributions

LM and S Zhang conceived and designed the study. MW, LC, YC, QG, and SZ performed all experiments. RW, SG, and S Zhu helped to collect, reformat, and analyze the primary data. LC, RW, and LM drafted the manuscript. LM and S Zhang proofread and revised the manuscript. All authors approved the final version of the manuscript.

## Supporting information


**Fig. S1.** Survival analysis regarding different MGP expression level based on other GEO datasets.

**Fig. S2.** The correlation between expression of MGP and downstream genes.

**Table S1.** siRNA sequences used for gene knockdown.

**Table S2.** Primer sequences used in quantitative real‐time polymerase chain reaction (qRT–PCR).

**Table S3.** Primer sequences used in chromatin immunoprecipitation (ChIP) assay.
