# Supplementary figures and images for: Intracellular matrix Gla protein promotes tumor progression by activating JAK2/STAT5 signaling in gastric cancer
Source: Mol Oncol. 2020 Mar 16;14(5):1045–58. doi: 10.1002/1878-0261.12652 (PMC7191194; doi:10.1002/1878-0261.12652)

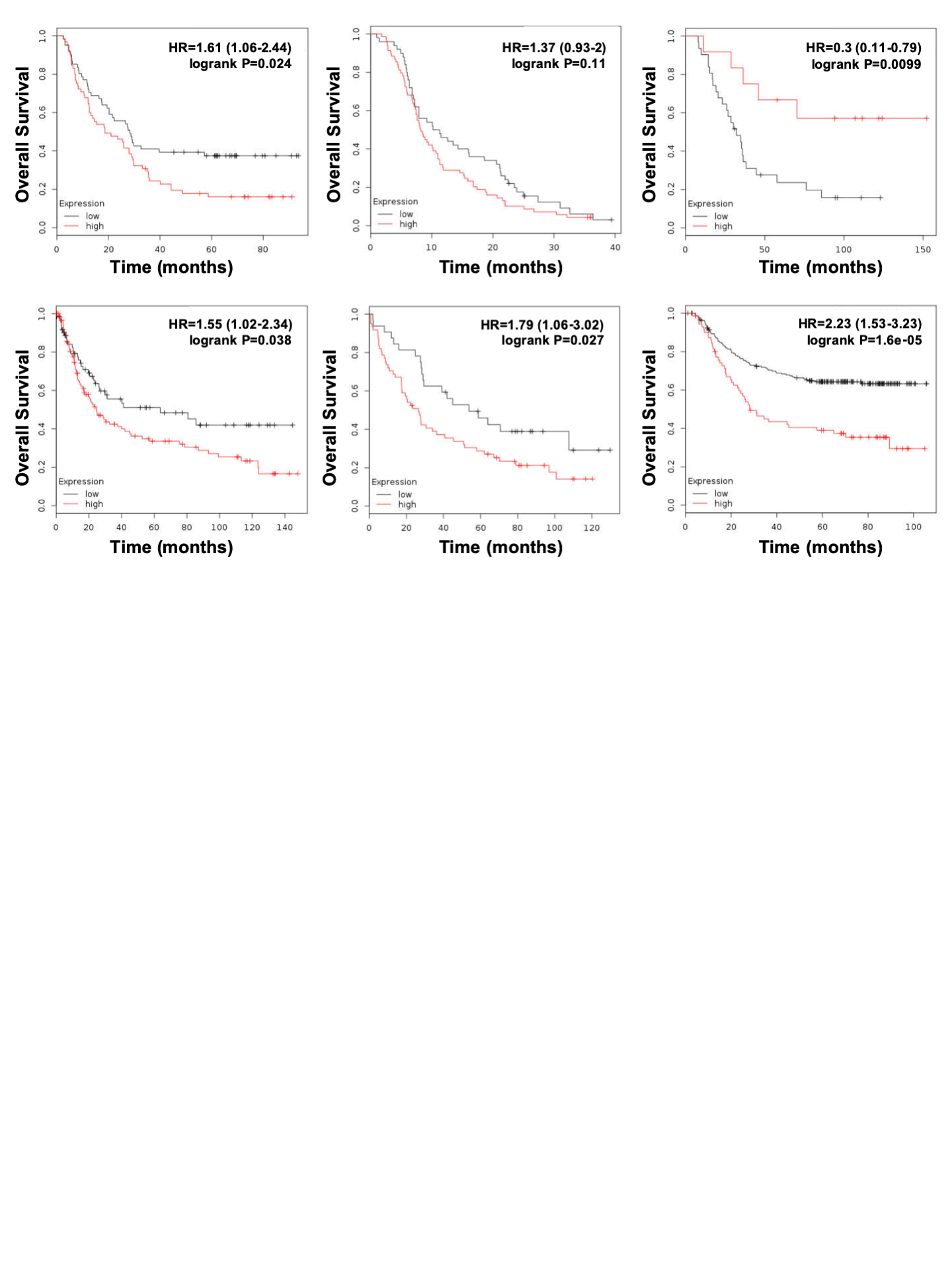

Supplement: Supplementary file 1 — Fig. S1. Survival analysis regarding different MGP expression level based on other GEO datasets. [file MOL2-14-1045-s001.tiff]

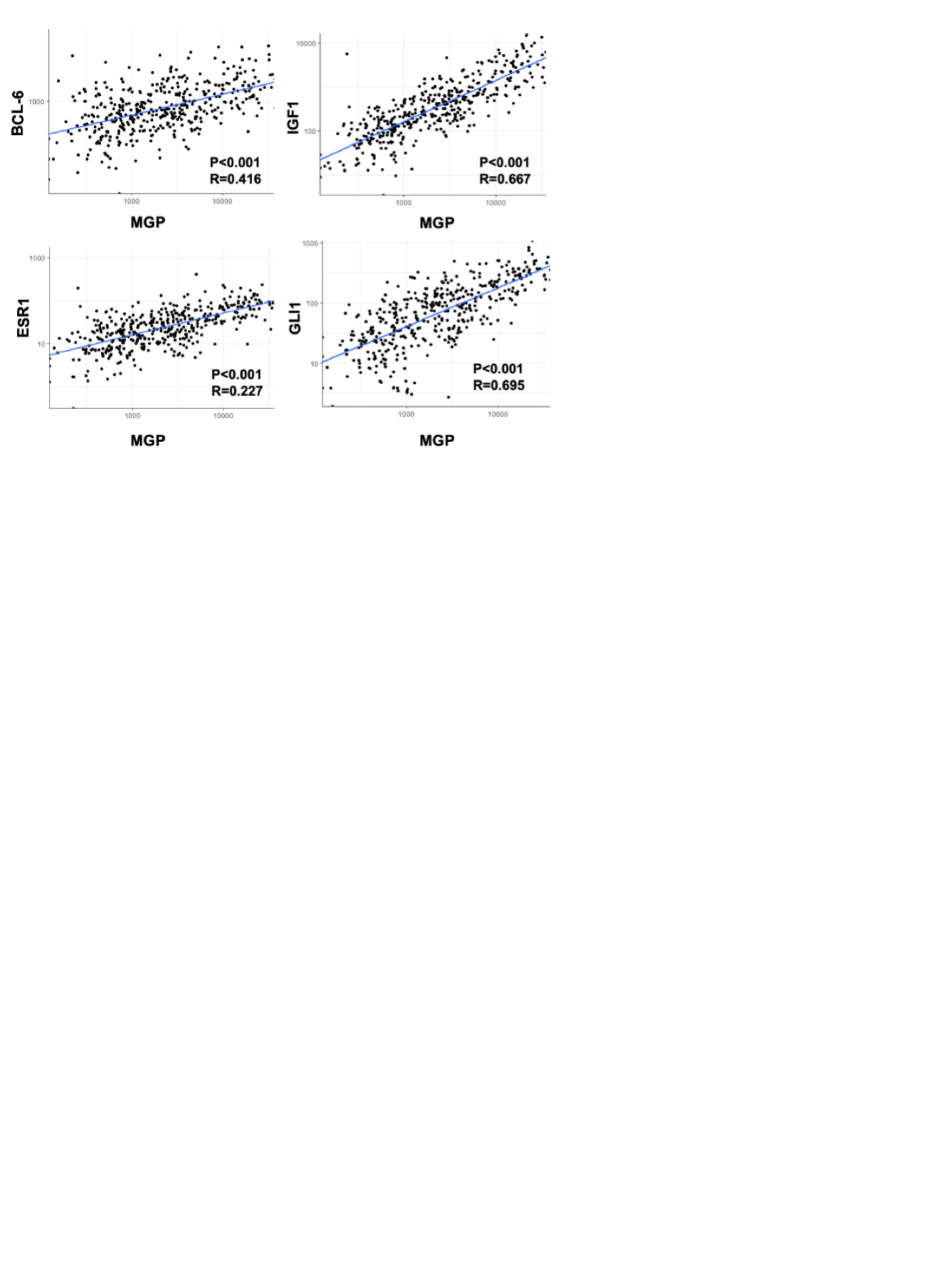

Supplement: Supplementary file 2 — Fig. S2. The correlation between expression of MGP and downstream genes. [file MOL2-14-1045-s002.tiff]
