# Supplementary material for: Intracellular matrix Gla protein promotes tumor progression by activating JAK2/STAT5 signaling in gastric cancer
Source: Mol Oncol. 2020 Mar 16;14(5):1045–58. doi: 10.1002/1878-0261.12652 (PMC7191194; doi:10.1002/1878-0261.12652)
Supplement: Supplementary file 3 — Table S1. siRNA sequences used for gene knockdown. [file MOL2-14-1045-s003.docx]

**Supplementary Table 1. siRNA sequences used for gene knockdown.**

| siRNA | Sequence |
| --- | --- |
| si-MGP#1 | F: 5’-CCCUACUGCUGCUACACAATT-3’ |
|  | R: 5’-UUGUGUAGCAGCAGUAGGGTT-3’ |
| si-MGP#2 | F: 5’-GAUAAGUAAUGAAAGUGCATT-3’ |
|  | R: 5’-UGCACUUUCAUUACUUAUCTT-3’ |
| si-Ctrl | F: 5’-UUCUUCGAACGUGUCACGUTT-3’ |
|  | R: 5’-ACGUGACACGUUCGGAGAATT-3’ |

*Note:* si-MGP, Matrix Gla protein small interfering RNA; si-Ctrl, negative control small interfering RNA.
