# Supplementary material for: Intracellular matrix Gla protein promotes tumor progression by activating JAK2/STAT5 signaling in gastric cancer
Source: Mol Oncol. 2020 Mar 16;14(5):1045–58. doi: 10.1002/1878-0261.12652 (PMC7191194; doi:10.1002/1878-0261.12652)
Supplement: Supplementary file 4 — Table S2. Primer sequences used in quantitative real‐time polymerase chain reaction (qRT–PCR). [file MOL2-14-1045-s004.docx]

**Supplementary Table 2. Primer sequences used in quantitative real-time polymerase chain reaction (qRT-PCR).**

| Gene | Sequence |
| --- | --- |
| MGP | F: 5’-AGAACGCTCTAAGCCTGTCCA-3’ |
|  | R: 5’-GGCAGCATTGTATCCATAAACC-3’ |
| BCL-2 | F: 5’-TGGTCGCCTTCGGATGTTG-3’ |
|  | R: 5’-CCTGTCTGTCCCTTAATGGTTTC-3’ |
| CCND2 | F: 5’-CTGTCTCTGATCCGCAAGCAT-3’ |
|  | R: 5’-GGTGGGTACATGGCAAACTTAAA-3’ |
| SOCS2 | F: 5’-TTAAAAGAGGCACCAGAAGGAAC-3’ |
|  | R: 5’-AGTCGATCAGATGAACCACACT-3’ |
| BCL-6 | F: 5’-ACACATCTCGGCTCAATTTGC-3’ |
|  | R: 5’-AGTGTCCACAACATGCTCCAT-3’ |
| GLI1 | F: 5’-GGGTGCCGGAAGTCATACTC-3’ |
|  | R: 5’-GCTAGGATCTGTATAGCGTTTGG-3’ |
| IGF1 | F: 5’-GCTCTTCAGTTCGTGTGTGGA-3’ |
|  | R: 5’-GCCTCCTTAGATCACAGCTCC-3’ |
| GAPDH | F: 5’-GGAGCGAGATCCCTCCAAAAT-3’ |
|  | R: 5’-GGCTGTTGTCATACTTCTCATGG-3’ |

*Note:* MGP, Matrix Gla protein; BCL-2, B-cell lymphoma 2; CCND2, cyclin D2; SOCS2, suppressor of cytokine signaling 2; BCL-6, B-cell lymphoma 6; GLI1, glioma-associated oncogene homolog 1; IGF1, insulin-like growth factor 1; GAPDH, glyceraldehyde-3-phosphate dehydrogenase.
