# Supplementary material for: Intracellular matrix Gla protein promotes tumor progression by activating JAK2/STAT5 signaling in gastric cancer
Source: Mol Oncol. 2020 Mar 16;14(5):1045–58. doi: 10.1002/1878-0261.12652 (PMC7191194; doi:10.1002/1878-0261.12652)
Supplement: Supplementary file 5 — Table S3. Primer sequences used in chromatin immunoprecipitation (ChIP) assay. [file MOL2-14-1045-s005.docx]

**Supplementary Table 3. Primer sequences used in chromatin immunoprecipitation (ChIP) assay.**

| Gene | Sequences |
| --- | --- |
| SOCS2 | F: 5’-TGTTCCTTTATCCCAGTCCC-3’ |
|  | R: 5’-AGGCACTTACCCTCTTTGGA-3’ |
| BCL-2 | F: 5’-GGTCCTGATACCCTAATCCTC-3’ |
|  | R: 5’-AGGTGCTCAACAGATGAATG-3’ |
| CCND2 | F: 5’-ACAAGGGCAGGAGGATTAGG-3’ |
|  | R: 5’-TCATTCTGTAGGTGTAGCACGC-3’ |

*Note:* SOCS2, suppressor of cytokine signaling 2; BCL-2, B-cell lymphoma 2; CCND2, cyclin D2.
